# Epidemiology of intimate partner violence perpetration and victimisation in a representative sample

**DOI:** 10.1017/S2045796023000069

**Published:** 2023-04-19

**Authors:** Vera Clemens, Jörg M. Fegert, Barbara Kavemann, Thomas Meysen, Ute Ziegenhain, Elmar Brähler, Andreas Jud

**Affiliations:** 1 Department for Child and Adolescent Psychiatry/Psychotherapy, University of Ulm, Germany; 2 Competence Center Child Protection in Medicine Baden-Württemberg, Ulm, Germany; 3 Social Sciences Research Institute on Gender Issues/FIVE, Freiburg, Germany; 4 International Centre for Socio-Legal Studies (SOCLES), Tübingen, Germany; 5 Department of Psychosomatic Medicine and Psychotherapy, University Medical Center Mainz of the Johannes Gutenberg University Mainz, Mainz, Rheinland-Pfalz DE 39068, Germany; 6 Integrated Research and Treatment Center Adiposity Diseases, Behavioral Medicine Research Unit, Department of Psychosomatic Medicine and Psychotherapy, University of Leipzig, Medical Center

**Keywords:** Epidemiology, gender differences, risk factors, trauma, violence

## Abstract

**Aims:**

Intimate partner violence (IPV) is a major global public health problem. Although IPV is known to be frequent and perpetration and victimisation often co-occur, large representative samples assessing both, male and female IPV perpetration and victimisation and overlaps are missing to date. Thus, we aimed to assess victimisation and perpetration and its overlap in physical, sexual, psychological and economic IPV in a representative sample of the German population.

**Methods:**

We conducted a cross-sectional, observational study in Germany from July to October 2021. Using different sampling steps including a random route procedure, a probability sample of the German population was generated. The final sample consisted of 2503 persons (50.2% female, mean age: 49.5 years). Participants were asked about socio-demographic information in a face-to-face interview and experience of physical, psychological, sexual and economic IPV using a questionnaire.

**Results:**

A significant proportion of persons in Germany reporting IPV are both perpetrator and victim for each IPV form. The biggest overlap between perpetration and victimisation was seen for psychological IPV. Major risk factors for IPV perpetration only were male gender and adverse childhood experiences (ACEs) while major risk factors for IPV victimisation only comprised of female gender, low household income and ACEs. In the perpetration and victimisation group, gender differences were less significant; older age and lower household income did increase the likelihood of combined perpetration and victimisation.

**Conclusions:**

We have identified a significant overlap of perpetration and victimisation of IPV in the German population for men and women. However, men are at much higher risk to perpetrate IPV without being a victim. Further research and the development of adapted approaches for contexts of overlapping IPV are necessary.

## Introduction

Intimate partner violence (IPV) is a major global public health problem (Ellsberg *et al*., 2008) with potentially detrimental outcomes for victims (Gerber *et al*., 2014). According to WHO, IPV ‘refers to behaviour by an intimate partner or ex-partner that causes physical, sexual or psychological harm, including physical aggression, sexual coercion, psychological abuse and controlling behaviours’ (WHO, 2021). The Council of Europe's Convention on preventing and combatting violence against women and domestic violence (better known as the Istanbul Convention) conceptualises it encompassing four dimensions: physical, sexual, psychological and economic violence (BMFSFJ, 2018). Physical violence includes behaviour such as slapping, hitting, kicking and beating; sexual violence includes e.g. forced sexual intercourse and other forms of sexual coercion (WHO, 2012). Psychological violence includes not only degradation or frightening but also containment from social relationships, work and social activities; while economic violence encompasses e.g. regulate access to finances and needed items such as clothes, food or a car (Moshtagh *et al*., 2023).

The reduction of IPV against women is claimed in the rights-based sustainable development goals (SDGs), that were adopted by all United Nations Member States in 2015 (UN, 2015). The WHO estimates a lifetime prevalence between 23 and 38% among ever-partnered women globally (World Health Organization WHO, 2013). In the European Union, a total of 43% of women have experienced some form of psychological violence by an intimate partner, of women who are or have been in a relationship with a man, 22% have experienced physical and or sexual violence (FRA – European Union Agency for Fundamental Rights, 2014). As national and international strategies have so far primarily focused on the reduction of violence against women, less data on IPV against men are available. A recent review reported prevalence rates between 3 and 20% for physical violence, between 0.2 and 7% for sexual violence and between 7 and 37% for psychological violence in partnerships against men (Kolbe and Büttner, 2020). Other international reviews and multi-country studies report similarly large prevalence rates (Stöckl *et al*., 2015; Jewkes *et al*., 2017). Importantly, literature suggests that many men affected by IPV as victims have been violent towards their partners themselves (Muelleman and Burgess, 1998; Lövestad and Krantz, 2012; Kolbe and Büttner, 2020). On the contrary, the majority of women perpetrating IPV have previously been victims of violence by their partners suggesting that a large part of IPV might be reciprocal and triggered by initial violence (Swan *et al*., 2012). Samples that assess both, female and male IPV perpetration and victimisation in the same sample are sparse. In a Swedish sample of 173 men and 251 women, perpetrator rates for physical IPV were comparable between women and men, while sexual abuse was perpetrated more frequently by men (Lövestad and Krantz, 2012). Schlack *et al*. (2013) reported in a nationally representative sample of 8152 participants higher perpetration of both physical IPV by women (1.3 *v.* 0.3% by men) and psychological IPV by women (3.8 *v.* 2.8% by men) in the last 12 months. Women had also affirmed increased 12-month prevalence of victimisation by both physical IPV (1.2%) and psychological IPV (6.1%) in comparison to men (0.9% victimisation by physical IPV and 3.3% victimisation by psychological IPV) (Schlack *et al*., 2013). While the authors discuss the assumption of female perpetration in self-defense and refer to other empirical literature reporting comparable rates of IPV perpetration for both women and men (Carney *et al*., 2007), the reliability and validity of this nationally representative survey for Germany are restricted as both perpetration of and victimisation by psychological and physical IPV were measured by a single item (Schlack *et al*., 2013). In the presence of mixed findings on the prevalence of IPV across genders, the debate about gender asymmetry or symmetry is ongoing (Chan, 2011). In his review, Chan (2011) highlights that although many studies point towards gender symmetry in prevalence, this results may be biased as men tend to under-report own IPV perpetration while women under-report own IPV victimisation. Moreover, there are some studies indicating a greater severity of physical IPV perpetrated by men compared to women (Chan, 2011). In line, official statistics show a clear gender gap in severe IPV encompassing dangerous physical injury, rape, murder and homicide with much higher rates of men being perpetrating (Bundeskriminalamt, 2021).

To date, large representative samples that include data on lifetime prevalence for both IPV victimisation and perpetration for both genders, analysing the overlap, are so far largely missing. This research gap highlights the need to explore and draft hypotheses on risk factors for overlapping experiences of victimisation and perpetration. Therefore, in a representative sample of the German population, we aimed to assess victimisation and perpetration and its overlap in physical, sexual, psychological and economic IPV. Additionally, we aimed to explore and identify differences in risk factors for those who are both victimised and perpetrate IPV in comparison to perpetration and victimisation without overlap. According to current literature, major risk factors for IPV victimisation comprise female gender, young age, poverty risk and victimisation in youth and childhood (Capaldi *et al*., 2012; Yakubovich *et al*., 2018). The second aim of our analysis was to assess whether these factors are relevant for not only victimisation but also perpetration of IPV in our population-based sample. Based on the emphasis on female victimisation of in policy and previous research, we assume females to be more likely victimised without perpetration compared to males.

## Methods

### Sample

For this observational approach, a representative sample of the German population was aimed to be generated by a research institute (USUMA, Berlin) via a three-step procedure. First, for systematic area sampling, geographic units in the entire inhabited area of Germany were sampled based on the municipal classification of the Federal Republic of Germany. Based on these geographic units, around 53 000 areas were delimited electronically, containing an average of around 700 private households each. Areas were then layered regionally according to districts into a total of around 1500 regional layers and then divided into 128 ‘networks’. Networks were then used as sampling frames, containing 258 single sample points proportionate to the distribution of private households in Germany each. In a second selection step, via a random route procedure, private households to be surveyed at each sample point were systematically selected: households of every third residence in a randomly selected street were invited to participate in the study. In a third step of random selection, in multi-person households, a kish-selection grid was used to ensure random participation. Selected participants had to be at least 14 years of age and had to have sufficient knowledge of the German language. Data collection took place between February and April 2020. Out of 5668 initially contacted households, 2503 persons completed the survey (response rate = 44.1%). Main reasons for non-participation were refusal to identify a target person within the selected household (23.5%, in relation to all contacted 5668 households), failure to contact anyone in the household after four attempts (13.4%) and refusal of the target person to participate (13.2%).

Individuals who agreed to participate were given information about the study and informed consent was obtained. In the case of minors, participants gave informed assent with informed consent being provided by their caregivers. Participants were informed that the study was about psychological health and well-being. First, socio-demographic information was collected using an interview format by the research staff. Next, the questionnaire and a sealable envelope were handed out. This questionnaire was self-completed due to the sensitive nature of the items. Research staff remained nearby in case the participants needed further information. The completed questionnaire was then linked to the respondent's socio-demographic data, but did not contain name, address or any other personal identifiers. Data on the final sample [50.2% female, average age of 49.5 years (s.d. = 17.5)] are given in Table 1.

**Table 1.** Sample characteristics

	Total 2503	Female 1256 (50.2)	Male 1247 (49.8)
Age, mean (s.d.), years	49.53 (17.51)	49.98 (17.58)	49.07 (17.43)
14–40	838 (33.5)	404 (32.2)	434 (34.8)
41–60	964 (38.5)	504 (40.1)	460 (36.9)
>60	700 (28.0)	348 (27.7)	352 (28.3)
German citizenship, *N* (%)	2409 (96.2)	1217 (96.9)	1192 (95.6)
Highest level of education, *N* (%)			
Left school before graduation	52 (2.1)	31 (2.5)	21 (1.7)
‘Hauptschulabschluss’ (year 9, lower secondary school certificate)	702 (28.5)	362 (29.2)	340 (27.8)
‘Mittlere Reife’ (year 10, middle secondary school certificate)	1112 (45.1)	567 (45.8)	545 (44.5)
A-levels/university degree	598 (24.3)	279 (22.5)	319 (26.0)
Equalised disposable household income, mean (s.d.), €	1839.38 (726.08)	1768.12 (692.75)	1910.57 (751.49)
<1000	268 (10.8)	151 (12.2)	117 (9.5)
1000–3000	1931 (78.1)	972 (78.6)	959 (77.5)
>3000	274 (11.1)	113 (9.1)	161 (13.0)
Number of experienced ACEs, *N* (%)			
0	1399 (57.9)	700 (57.7)	699 (58.1)
1–3	763 (31.6)	382 (31.5)	381 (31.7)
4–10	255 (10.6)	132 (10.9)	123 (10.2)

Presented as number (*n*) or mean value (*M*) and standard deviation (s.d.) and %.

### Ethical approval

The study was conducted in accordance with the Declaration of Helsinki, and was approved by the Ethics Committee of the Medical Department of the University of Leipzig (002/20-ek). The study fulfilled both, the ethical guidelines of the International Code of Marketing and Social Research Practice of the International Chamber of Commerce and of the European Society of Opinion and Marketing Research.

### Measures

Socio-demographic questions used for this study included age, gender and socio-economic status. Importantly, data from women and men are coming from the above described sampling approach aiming for a representative sample and are independent and not dyadic (coming from couples where both partners were questioned). Gender was assessed in a binary form; age of the respondents was collapsed into three categories (14–40, 41–60, 61+ years) to roughly allow for a comparison between different generations associated with different perspectives on IPV. School graduation was assessed in four groups: no-school graduation, A levels, middle maturity and secondary school certificate, according to the German school system. Equalised household income was calculated in dependence of the number of persons living in the household and categorised into the groups >3000 euros, 1000–3000 euros and <1000 euros. Having less than 1000 euros equalised household income monthly was defined as a poverty risk.

Prevalence rates of physical, sexual, psychological and economic IPV were assessed using the questionnaire of the UN Multi-Country Study on Men and Violence (Jewkes *et al*., 2017). Items were translated and validated in group discussions. They were further adapted to address both violence against women and men, as described before (Jud *et al*., 2023). IPV items were grouped into psychological violence (five items), economical violence (three items), physical violence (three items) and sexual violence (four items). Note that this questionnaire is a screening instrument developed to measure the epidemiology of certain incidents. It does not represent latent constructs; incidents grouped under the same category are therefore not conceptualised to necessarily co-occur. They rather represent variations of potential types of violence. Consequently, the Cronbach's *α* for subscales ranging from *α* = 0.51 (economical IPV) to *α* = 0.88 (sexual IPV) do not and cannot achieve the same levels expected for conceptual measures. While we have used a previously established questionnaire to allow for international comparison, validity indicators for this questionnaire are unfortunately lacking – a characteristic that also applies to other short questionnaires used to measure the epidemiology of IPV in the general population.

Adverse childhood experiences (ACEs) were assessed with the Adverse Childhood Experiences Questionnaire, a standard tool for the retrospective assessment of ACEs (Felitti *et al*., 1998). The German version of the questionnaire has a satisfactory reliability (Cronbach's *α* = 0.76) (Wingenfeld *et al*., 2011). ACEs comprise according to the original publication by Fellitti child maltreatment, in detail physical, emotional and sexual abuse, physical as well as physical and emotional neglect and household dysfunctions, comprising separation of a parent, the experience of parental IPV, growing up in a household with a person with a mental disorder or abusing substances and having one incarcerated parent (Felitti *et al*., 1998). For our analyses, instead of summing up ACEs to a score between 0 and 10, two separate sum scores (each 0–5) were calculated – one for child maltreatment and one for household dysfunctions. As literature shows that although there is a strong dose-dependency between the number of experienced ACEs and numerous outcomes including mental and somatic health (Felitti *et al*., 1998; Clemens *et al*., 2018), and violent behaviour (Witt *et al*., 2019), splitting ACEs between child maltreatment and household dysfunctions has been the original conceptualisation of the instrument and makes sense giving (1) a better insight into the interplay between ACEs (Clemens *et al*., 2019) and (2) as simply summing up the ACE score gives the same weight e.g. to the experience of sexual abuse and parental separation (McLennan *et al*., 2020).

### Statistical analyses

All analyses were conducted with SPSS, version 21. Prevalence rates were determined by descriptive analyses. In order to compare the four groups: no IPV, victimisation without perpetration, perpetration without victimisation and perpetration and victimisation, inferential statistics were modelled as multinomial logistic regression analyses separately for each of the four assessed IPV forms (physical, sexual, psychological and economic IPV). Gender (binary, female/male, reference: female), household income (>3000, 1000–3000 and <1000 euros, reference: <1000 euros), age (14–40, 41–60, >60 years, reference: 14–40 years), ACE scores (separately for CM; ⩾2 forms, 1 form, 0 form, reference: 0 form) and household dysfunction (⩾1 forms, 0 form, reference: 0 form) and highest graduation level (A level, middle maturity, secondary school certificate, no-school graduation, reference: no-school graduation) were included as determinants. Results are displayed as model statistics (LR-chi^2^, pseudo-*R*^2^) and relative risk ratio (RRR) for determinants. Statistics are based on all cases with valid data for all variables in the model.

## Results

### Prevalence rates of IPV perpetration, victimisation and combined perpetration and victimisation

To increase readability of findings, we have labelled victimisation without perpetration ‘victimisation only (VO)’ and perpetration without victimisation ‘perpetration only (PO)’. Psychological IPV was reported most frequently by a total of by 247 (10.0%) of participants, followed by sexual IPV; reported by 235 (9.5%) participants. As rarest form, physical IPV was reported by 144 (5.8%) participants. In all four types of IPV, women were more prevalent in the victim only group, with the highest rate in sexual IPV [women: VO 194 (15.6%); men: VO 41 (3.3%), *p* < 0.001]. At the same time, men reported more frequently to be in the perpetrator only group in all four forms of IPV, with the highest rate in psychological IPV [men: PO 112 (9.1%)]. In the victimisation and perpetration group, prevalence rates were more comparable between women and men (see Table 2).

**Table 2.** Perpetration and victimisation, separated for gender

	Female	Male	Total	Chi^2^	*p*-value
Psychological IPV					
Victimisation only	160 (12.9)	87 (7.1)	247 (10.0)		
Perpetration only	62 (5.0)	112 (9.1)	174 (7.0)		
Victimisation and perpetration	511(41.2)	507 (41.2)	1018 (41.2)	36.24	<0.001
Physical IPV					
Victimisation only	100 (8.0)	44 (3.6)	144 (5.8)		
Perpetration only	14 (1.1)	24 (1.9)	38 (1.5)		
Victimisation and perpetration	33 (2.7)	30 (2.4)	63 (2.5)	25.19	<0.001
Economical IPV					
Victimisation only	164 (13.2)	54 (4.4)	218 (8.8)		
Perpetration only	17 (1.4)	43 (3.5)	60 (2.4)		
Victimisation and perpetration	36 (2.9)	20 (1.6)	56 (2.3)	75.39	<0.001
Sexual IPV					
Victimisation only	194 (15.6)	41 (3.3)	235 (9.5)		
Perpetration only	20 (1.6)	112 (9.1)	132 (5.3)		
Victimisation and perpetration	42 (3.4)	35 (2.8)	77 (3.1)	165.86	<0.001

Presented as *n* (%).

### Predictors for perpetration, victimisation and combined perpetration and victimisation of IPV

Male gender was associated with higher odds for perpetration only (PO) and lower odds for victimisation only (VO) of psychological violence (PO: 1.75, *p* = 0.002; VO: 0.53, *p* < 0.001), economic violence (PO: 2.27, *p* = 0.006; VO: 0.29, *p* < 0.001) and sexual violence (PO: 5.15, *p* < 0.001). Additionally, male gender was associated with lower odds for VO of physical violence (0.44, *p* < 0.001).

Belonging to the age group of 41–60 years was associated with higher odds for VO of psychological violence (1.90, *p* < 0.001) and perpetration and victimisation (PaV) of psychological violence (2.03, *p* < 0.001).

Household income above €3000 predicted lower odds for VO and PaV of psychological violence (VO: 0.36, *p* = 0.003; PaV: 0.54, *p* = 0.004) and lower odds for VO of sexual violence (0.45, *p* = 0.019 for income >€3000 and 0.65, *p* = 0.042 for income of €1000–3000). Regarding economical violence, a household income above €3000 (0.32, *p* = 0.002) as well as a household income between €1000 and €3000 (0.50, *p* = 0.001) was associated with lower odds for VO.

The experience of child maltreatment was associated with strongly increased odds for VO; PO as well as combined PaV of all types of IPV. The experience of household dysfunction was associated with increased odds for VO PO, and PaV of all types of sexual violence, with VO of physical IPV, with VO and PO of economic violence and strongly increased odds for VO, PO as well as PaV of psychological violence (see Tables 3–6).

**Table 3.** Predictors for perpetrator, victim and perpetrator and victim based on psychological violence

		Victimisation only				Perpetration only				Perpetration and victimisation			
		RRR^a^	*p*-value	95% CI		RRR^a^	*p*-value	95% CI		RRR^a^	*p*-value	95% CI	
Gender	Male	0.53	<0.001	0.39	0.71	1.75	0.002	1.23	2.49	1.00	0.992	0.83	1.21
Household income	>€3000	0.36	0.003	0.18	0.70	1.32	0.455	0.63	2.77	0.54	0.004	0.35	0.82
	€1000–3000	0.74	0.187	0.48	1.16	1.25	0.453	0.69	2.26	0.99	0.935	0.71	1.36
Age	⩾61 years	1.32	0.182	0.88	1.98	1.35	0.165	0.88	2.06	1.54	0.001	1.19	1.98
	41–60 years	1.90	<0.001	1.34	2.70	1.01	0.950	0.66	1.55	2.03	<0.001	1.61	2.55
Child maltreatment score	⩾2 forms of CM	3.47	<0.001	2.13	5.66	3.90	<0.001	2.27	6.72	4.16	<0.001	2.91	5.94
	1 form of CM	1.76	0.038	1.03	3.01	3.16	<0.001	1.85	5.40	2.92	<0.001	2.07	4.11
Household dysfunction score	⩾1 form of HD	1.59	0.007	1.13	2.24	1.67	0.010	1.13	2.47	1.94	<0.001	1.56	2.43
	1 form of HD	0.58	0.248	0.23	1.46								
Highest graduation	A levels	0.42	0.063	0.17	1.05	0.58	0.353	0.18	1.83	1.17	0.703	0.52	2.61
	Middle maturity	0.42	0.063	0.17	1.05	0.46	0.182	0.15	1.43	1.01	0.985	0.46	2.22
	Secondary school certificate	0.53	<0.001	0.39	0.71	0.63	0.427	0.20	1.96	0.92	0.835	0.41	2.04

LR-chi^2^ = 344.74, pseudo-*R*^2^ = 0.15.

a An RRR >1 corresponds to a higher probability of perpetration only, victimisation only or perpetration and victimisation in comparison to respondents without any experience of IPV; comparison categories from top to bottom: gender female, household income <€1000, age: 14–40 years, child maltreatment and household dysfunction score: 0 ACEs, graduation: no-school graduation.

**Table 4.** Predictors for perpetrator, victim and perpetrator and victim based on economic violence

		Victimisation only				Perpetration only				Perpetration and victimisation			
		RRR^a^	*p*-value	95% CI		RRR^a^	*p*-value	95% CI		RRR^a^	*p*-value	95% CI	
Gender	Male	0.29	<0.001	0.21	0.41	2.27	0.006	1.26	4.10	0.51	0.019	0.29	0.90
Household income	>€3000	0.32	0.002	0.16	0.66	1.30	0.671	0.39	4.35	0.85	0.772	0.28	2.56
	€1000–3000	0.50	0.001	0.33	0.74	1.30	0.563	0.53	3.20	0.66	0.289	0.31	1.42
Age	⩾61 years	1.47	0.061	0.98	2.21	1.56	0.218	0.77	3.17	1.47	0.277	0.73	2.95
	41–60 years	1.08	0.674	0.74	1.58	1.27	0.487	0.64	2.51	0.90	0.769	0.45	1.80
Child maltreatment score	⩾2 forms of CM	2.45	<0.001	1.65	3.64	2.42	0.013	1.21	4.86	2.20	0.037	1.05	4.61
	1 form of CM	1.87	0.007	1.19	2.94	2.48	0.017	1.17	5.24	2.11	0.064	0.96	4.65
Household dysfunction score	⩾1 form of HD	2.73	<0.001	1.93	3.86	2.37	0.006	1.28	4.39	1.57	0.153	0.85	2.92
Highest graduation	A levels	0.68	0.393	0.28	1.66	0.33	0.117	0.08	1.32	0.41	0.282	0.08	2.09
	Middle maturity	0.72	0.460	0.30	1.71	0.32	0.092	0.08	1.21	0.72	0.676	0.16	3.32
	Secondary school certificate	0.76	0.532	0.32	1.81	0.49	0.292	0.13	1.83	0.78	0.750	0.17	3.59

LR-chi^2^ = 249.41, pseudo-*R*^2^ = 0.16.

a An RRR >1 corresponds to a higher probability of perpetration only, victimisation only or perpetration and victimisation in comparison to respondents without any experience of IPV; comparison categories from top to bottom: gender female, household income <€1000, age: 14–40 years, child maltreatment and household dysfunction score: 0 ACEs, graduation: no-school graduation.

**Table 5.** Predictors for perpetrator, victim and perpetrator and victim based on physical violence

		Victimisation only				Perpetration only				Perpetration and victimisation			
		RRR^a^	*p*-value	95% CI		RRR^a^	*p*-value	95% CI		RRR^a^	*p*-value	95% CI	
Gender	Male	0.44	<0.001	0.30	0.64	1.55	0.205	0.79	3.06	0.80	0.399	0.47	1.35
Household income	>€3000	0.74	0.437	0.34	1.60	1.24	0.701	0.41	3.72	1.06	0.913	0.35	3.23
	€1000–3000	0.78	0.342	0.47	1.30	0.44	0.053	0.19	1.01	0.95	0.904	0.45	2.04
Age	⩾61 years	1.04	0.876	0.63	1.71	1.18	0.727	0.47	2.97	0.60	0.169	0.29	1.24
	41–60 years	1.15	0.533	0.74	1.78	1.50	0.340	0.65	3.48	0.81	0.492	0.44	1.48
Child maltreatment score	⩾2 forms of CM	3.88	<0.001	2.48	6.08	2.60	0.026	1.12	6.02	8.41	<0.001	4.09	17.27
	1 form of CM	1.68	0.072	0.95	2.97	1.63	0.358	0.58	4.61	6.68	<0.001	3.22	13.85
Household dysfunction score	⩾1 form of HD	3.32	<0.001	2.13	5.16	2.01	0.073	0.94	4.31	1.68	0.104	0.90	3.16
Highest graduation	A levels	1.10	0.854	0.38	3.19	0.17	0.025	0.04	0.80	2.44	0.402	0.30	19.54
	Middle maturity	0.76	0.602	0.27	2.14	0.26	0.055	0.06	1.03	1.79	0.580	0.23	14.12
	Secondary school certificate	0.81	0.690	0.28	2.30	0.47	0.269	0.12	1.79	2.43	0.398	0.31	19.18

LR-chi^2^ = 240.50, pseudo-*R*^2^ = 0.17. aAn RRR > 1 corresponds to a higher probability of perpetration only, victimisation only or perpetration and victimisation; comparison categories from top to bottom: gender female, household income <€1000, age: 14–40 years, child maltreatment and household dysfunction score: 0 ACEs, graduation: no-school graduation.

**Table 6.** Predictors for perpetrator, victim and perpetrator and victim based on sexual violence

		Victimisation only				Perpetration only				Perpetration and victimisation			
		RRR^a^	*p*-value	95% CI		RRR^a^	*p*-value	95% CI		RRR^a^	*p*-value	95% CI	
Gender	Male	0.20	<0.001	0.14	0.29	5.15	<0.001	3.12	8.51	0.70	0.145	0.44	1.13
Household income	>€3000	0.45	0.019	0.23	0.88	0.79	0.588	0.33	1.87	1.42	0.499	0.51	3.96
	€1000–3000	0.65	0.042	0.43	0.98	1.19	0.585	0.64	2.20	1.27	0.547	0.58	2.77
Age	⩾61 years	1.12	0.576	0.75	1.68	1.12	0.653	0.69	1.81	0.89	0.714	0.47	1.68
	41–60 years	1.08	0.678	0.75	1.55	0.94	0.800	0.60	1.48	0.93	0.802	0.53	1.62
Child maltreatment score	⩾2 forms of CM	3.62	<0.001	2.43	5.39	3.13	<0.001	1.91	5.14	5.61	<0.001	3.08	10.21
	1 form of CM	3.16	<0.001	2.07	4.81	2.72	<0.001	1.60	4.61	2.81	0.005	1.38	5.73
Household dysfunction score	⩾1 form of HD	2.06	<0.001	1.47	2.88	2.19	<0.001	1.44	3.34	2.03	0.012	1.17	3.53
Highest graduation	A levels	1.16	0.761	0.44	3.12	2.43	0.254	0.53	11.14	0.91	0.888	0.24	3.43
	Middle maturity	1.49	0.413	0.57	3.87	1.40	0.667	0.31	6.37	0.85	0.806	0.24	3.08
	Secondary school certificate	1.18	0.733	0.45	3.10	1.58	0.552	0.35	7.23	0.72	0.623	0.20	2.66

LRchi^2^ = 411.06, pseudo-*R*^2^ = 0.22.

a An RRR >1 corresponds to a higher probability of perpetration only, victimisation only or perpetration and victimisation in comparison to respondents without any experience of IPV; comparison categories from top to bottom: gender female, household income <€1000, age: 14–40 years, child maltreatment and household dysfunction score: 0 ACEs, graduation: no-school graduation.

Educational level was not associated with victimisation or perpetration of any IPV form.

## Discussion

This is the first large representative study in Germany assessing both, perpetration and victimisation and the overlap of both of all four types of IPV in women and men. Regarding our main research question, to assess victimisation and perpetration and its overlap in physical, sexual, psychological and economic IPV, results show a significant proportion of persons who are both perpetrator and victim for each IPV form. The biggest overlap between perpetration and victimisation was seen for psychological IPV. Focusing on gender, while men were more likely to be perpetrator only, women were more likely to be victim only in all four assessed types of IPV. Prevalence rates were more comparable among both males and females in the perpetration and victimisation groups. Male gender was the main risk factor for perpetration only and was associated with decreased likelihood for victimisation only. Thus, our data show for the first time that there is a big overlap of IPV perpetration and victimisation in a representative sample in males and females, with males being at greater risk for perpetration only and females at greater risk for victimisation only.

This is in line with literature: in a Swedish population-based study, for example, lifetime exposure to physical assault was higher in women compared to men while men reported more frequently physical violence against their partner than women (Lövestad and Krantz, 2012). Women were also more commonly victim of sexual violence while men were more frequently perpetrator. In this study by Lövested and Krantz, 63.9% of men exposed to physical or sexual violence were also perpetrator while this was only the case for 39.4% of women (Lövestad and Krantz, 2012). As literature suggests men to tend to under-report own IPV perpetration while women under-report own IPV victimisation (Chan, 2011), the reported discrepancy between men and women in this study may in fact even be larger with higher rates of perpetration only in men and victimisation only in women.

Based on our results, self-identified perpetration of psychological violence is frequent in females; the overlap between victimisation and perpetration is largest for this subtype. It seems as psychological IPV is largely reciprocal. However, these quantitative data do not provide information about defensive or offensive use of psychological violence (Jud *et al*., 2023). Sexual violence is at the other end of the spectrum, with the smallest overlap between victimisation and perpetration and the most clear-cut gender difference: perpetration is primarily male; women are primarily victims of sexual violence. Regarding the experience of both, perpetration and victimisation of IPV, no gender effect was seen in our data despite of economic violence. Consequently, while men are much more likely to perpetrate IPV without being victim of IPV, perpetration with victimisation seems to be of similar frequency in men and women, in line with literature (Schlack *et al*., 2013). In a sample of 412 women who had used physical aggression against their male partners, 92% had experienced some form of physical and/or sexual victimisation from their male partners (Swan *et al*., 2012), underlining the hypothesis reciprocal character of IPV in women. However, as our study has a cross-sectional design, chronological order of victimisation and perpetration cannot be deduced. Data examining this interplay between perpetration and victimisation of IPV in population-based samples are missing to date. Prospective studies and dyadic samples are needed to improve understanding on the interplay between IPV victimisation and perpetration in men and women.

The second aim of our study was to assess risk factors for IPV victimisation and perpetration. Focusing on risk factors, the highest odds for perpetration only, victimisation only and both, perpetration and victimisation, of all forms of IPV were seen in dependence of the experience of ACEs, particularly multiple child maltreatment victimisation or multiple household dysfunctions in childhood. Having experienced two and more forms of child maltreatment doubled, tripled or even quadrupled the relative risk for victimisation, perpetration and combined victimisation for all forms of IPV. The more than eight-fold increased risk for perpetration only of physical abuse in the presence of two and more child maltreatment forms was even more exceptional. Our study thus confirms previous evidence on childhood adversity as a major risk factor for the experience of IPV; the dose–response relationship has also regularly been confirmed. In a recent review on risk factors for IPV against men, 10–40% of affected men reported having been abused or maltreated as children (Kolbe and Büttner, 2020). In the United Nations Multi-country Study on Men and Violence in Asia and the Pacific, including 3106 women, sexual, physical and emotional abuse was reported more frequently by women with lifetime experience of all forms of IPV (Jewkes *et al*., 2017). Moreover, the association between ACEs and IPV perpetration is well known from literature (Mair *et al*., 2012; Lee *et al*., 2022). Among the generally strong associations, our data suggest a particularly strong connection between child maltreatment experience and physical IPV perpetration. Pathways for this strong connection – e.g. via inhibited anger management – need to be explored in future studies.

Our data suggest a higher equalised household income to be protective against victimisation by psychological, sexual and economical violence – the relative risk was reduced from around 50% to around three-thirds. Somewhat surprisingly, the decreased relative risk for victimisation by physical violence was not significant. Income loss and economic hardship can lead to feelings of economic stress and consequent marital conflict (Elder, 1974; Elder and Conger, 2000). Strengthening financial security in households has already been proposed as one step in IPV prevention (Centers for Disease Control Prevention, 2017). Still, given the fact that Germany is a high-income country, the marked relevance of income in this study is surprising. Literature suggests that particularly the subjective perceptions of economic insecurity may be a greater influencing factor for family violence than actual objective concern (Lee *et al*., 2013; Schneider *et al*., 2017). As much of data collection for the survey took place during the first COVID-19-associated lockdown in Germany, the perception of upcoming recession and economic hardship may have played a role particularly in families with lower income. However, decreased readiness to disclose economic IPV in higher socio-economic strata cannot be excluded and may limit the validity of this finding. Interestingly, income was not associated with the risk of physical IPV. This finding contradicts previous literature showing a robust link between low socio-economic status, poverty and IPV perpetration and victimisation (Fox *et al*., 2002; Ahmadabadi *et al*., 2020). However, literature also suggests that a disequilibrium of income between partners is a relevant risk for IPV (Ahmadabadi *et al*., 2020). In our study, only total household income was assessed, this issue of operationalisation may affect our results.

Age of the respondents was significantly associated with victimisation only and perpetration only for the experience of psychological violence. The odds for both psychological victimization only and perpetration and victimization were highest in the generation of middle-aged people; the relative risk was (almost) doubled to belong to one of the two groups if respondents in the middle-age group. While this is partly in accordance with literature (Sanz-Barbero *et al*., 2019), it is still surprising given the measurement of lifetime prevalence of IPV. This means, older age gives more possibility to accumulate violent experiences. However, the difference might also be due to an increased readiness in younger generations to perceive psychologically violent acts as such as consequence of the increased awareness. Taken together, our data show for the first time risk factors for both, IPV perpetration and victimisation, in a representative sample. These risk factors comprise childhood adversity for IPV perpetration and victimisation, low income as risk factor for IPV victimisation only and middle age as risk factor for psychological IPV.

The major advantage of this study is the representativeness of the sample. This allows controlling for major socio-demographic confounders and ensures a high generalisability of the results. The assessment of perpetration and victimisation of all four forms of IPV enables a broad insight into the prevalence of IPV in Germany. The major limitation is the cross-sectional character of survey. Chronological order of victimisation and perpetration of IPV cannot be deduced. Second, the results on IPV and ACEs are based on self-report and therefore prone to biases in reporting: social norms, attitudes on masculinity or femininity might contribute to differences between reported and actual experience of IPV (Próspero, 2008). While Jud *et al*. (2023) highlight in the present survey that women are more regularly victimised and more often victimised by multiple forms of IPV, additional information on severity or chronicity of IPV is lacking and should be explored in future research. As gender was assessed in a binary form, no information can be given to experiences of IPV of non-binary persons.

## Conclusion

Focusing on the elimination of IPV, as claimed in the UN SDGs (UN, 2015), is a major challenge that should be addressed urgently facing the here reported high prevalence rates. The identified overlap of perpetration and victimisation of IPV gives a major new insight into IPV patterns. Particularly the fact that men are at much higher risk to perpetrate IPV without being a victim of IPV while perpetration and victimisation of IPV at the same time are of similar frequency in men and women should be addressed in further research. As an implication, the results call for investigation into developing adapted approaches of prevention and intervention for contexts of overlap between victimisation and perpetration and for perpetration without victimisation.

Longitudinal in-depth studies may answer the question whether IPV perpetration of women has a more reactive character compared to men. Furthermore, the issues of overlap in the prevalence of IPV have to be more strongly connected to indicators of severity or chronicity. While Chan (2011) highlights an overview of gender differences in IPV that ‘men and women may exhibit similar rates of IPV when no contexts, motivations, and consequences are considered’, evidence so far points to direction of men perpetrating more regularly and more severe IPV. Ultimately, national crime statistics highlight the elevated rates of femicides (Bundeskriminalamt, 2021). Our data also point out the relevance of economic situation and the experience of childhood adversity for psychological, sexual and psychological IPV, but, contrary to previous literature, not for physical IPV. Future research should therefore scrutinise the potentially differential associations between economic support and risk for different types of IPV while investing into the measurement of poverty indicators (Blumenthal and Rothwell, 2018). Still, economic support, on top of other positive outcomes, may be instrumental in also partially reducing the striking high rates of IPV.

As the experience of childhood adversity is a significant predictor for all forms of IPV, targeted approaches including offers for conflict management in partnerships for individuals who have experienced ACEs are needed.

## Data Availability

The datasets generated during the current study are not publicly available due to conditions on participant consent.
